# Repellent and Deterrent Oviposition Activity of Essential Oils from the Native Chilean Plant *Drimys winteri* Against *Drosophila suzukii* (Diptera: Drosophilidae)

**DOI:** 10.3390/plants15111676

**Published:** 2026-05-29

**Authors:** Jocelyne Tampe, Dante Sandoval, Javier Espinoza, Camila Ruiz, Andrés Quiroz, Mónica Rubilar

**Affiliations:** 1 Departamento de Ingeniería Química, Facultad de Ingeniería y Ciencias, Universidad de La Frontera, Avenida Francisco Salazar 01145, Casilla 54-D, Temuco 4811230, Chile; dante.sandoval1903@gmail.com (D.S.); c.ruiz06@ufromail.cl (C.R.); 2 Laboratorio de Química Ecológica, Departamento de Ciencias Químicas y Recursos Naturales, Universidad de La Frontera, Avenida Francisco Salazar 01145, Casilla 54-D, Temuco 4811230, Chile; javier.espinoza@ufrontera.cl (J.E.); andres.quiroz@ufrontera.cl (A.Q.); 3 Centro de Investigación Biotecnológica Aplicada al Medio Ambiente (CIBAMA), Universidad de La Frontera, Avenida Francisco Salazar 01145, Casilla 54-D, Temuco 4811230, Chile; 4 Doctorado en Ciencias de Recursos Naturales, Facultad de Ciencias Químicas y Recursos Naturales, Universidad de La Frontera, Avenida Francisco Salazar 01145, Casilla 54-D, Temuco 4811230, Chile; 5 Scientific and Technological Bioresource Nucleus, BIOREN, Universidad de La Frontera, Avenida Francisco Salazar 01145, Casilla 54-D, Temuco 4811230, Chile

**Keywords:** spotted wing fly, canelo, choice bioassays

## Abstract

*Drosophila suzukii* is one of the most destructive pests of soft fruits worldwide due to its high reproductive capacity, wide host range, and great adaptability. In this context, *Drimys winteri*, a tree native to southern Chile and Argentina, is recognized as a source of bioactive compounds with insecticidal and repellent properties. This study evaluated the repellent and oviposition-deterrent activity of essential oils (EOs) from the bark and leaf of *D. winteri* on *D. suzukii*. Chemical analysis by GC/MS showed that both EOs were dominated by monoterpenes, with α-pinene, β-pinene, and D-limonene being the major compounds, while the leaf EO exhibited greater chemical diversity and a higher proportion of sesquiterpenes. In choice bioassays, all treatments generated significant avoidance responses, with a preference for the control. The leaf EO showed the greatest repellent effect, exceeding 85% at 12 h and remaining above 80% at 96 h. In oviposition assays, the leaf EO significantly reduced egg laying at all concentrations, with negative oviposition preference index values indicating a consistent deterrent effect. Overall, the leaf EO of *D. winteri* showed repellent and oviposition-deterrent effects against *D. suzukii* under laboratory conditions.

## 1. Introduction

*Drosophila suzukii* (Matsumura) (Diptera: Drosophilidae), commonly known as the spotted wing drosophila (SWD), has emerged as one of the most destructive insect pests affecting fruit production globally. Native to Southeast Asia, it was first reported in Europe and North America in 2008 [1] and later detected in South America in 2013 [2]. In Chile, it was officially detected in 2017, when specimens were captured in traps placed in blackberry orchards along the Villarrica–Pucón International Highway, in the La Araucanía Region. This finding was confirmed by the Agricultural and Livestock Service (Servicio Agrícola y Ganadero—SAG), which prompted the implementation of national phytosanitary measures to prevent its spread [3].

The global invasion success of *D. suzukii* is largely attributed to its high ecological adaptability, reproductive capacity, broad host range, a wide variety of soft-skinned fruits, and strong passive and active dispersal abilities. The species can overwinter for extended periods and survive intercontinental transport in various developmental stages, including egg, larva, and adult, inside infested fruit or shipping containers [1,4,5,6,7,8]. Economically, the impact has been considerable. In the province of Trento, Italy, De Ros et al. [9] estimated losses of €3.3 million in crops, such as cherries, strawberries, raspberries, blueberries, and blackberries. In the United States, significant losses have also been reported. In Maine, in the northeastern part of the country, Yeh et al. [10] estimated losses of up to US$6.8 million in the blueberry industry, while in Minnesota, the raspberry industry reports annual losses of approximately US$2.35 million [11]. In South America, Benito et al. [12] estimated losses ranging from US$7.8 million in figs to US$21.4 million in peaches in Brazil. In Chile, although national-level losses have yet to be fully quantified, Buzzetti [13] reported losses of 10–15% of cherry production (1–2.7 t/ha), equivalent to US$5000–17,550/ha depending on the variety. In blueberries, damage affects approximately 8% of the fruit (1–1.5 t/ha), corresponding to losses of around US$4000/ha, with the potential to increase as pest pressure rises. In response, the Chilean government launched a two-year national project in 2021, investing CLP 360 million (approximately USD 493,000) to control this invasive pest in central production areas [14].

Current control strategies for *D. suzukii* are grounded in an integrated pest management (IPM) approach, which combines chemical, cultural, biological, and physical methods [15]. However, the heavy reliance on broad-spectrum synthetic insecticides presents several challenges. These compounds can negatively impact non-target organisms, including pollinators and beneficial arthropods; they may drive the evolution of pesticide-resistant populations and they raise concerns about chemical residues in fruit destined for export [9,16]. Moreover, these treatments are typically limited to managed crop areas, enabling rapid reinfestation from adjacent, untreated vegetation [17,18]. These limitations highlight the need for safer, eco-friendly pest control alternatives that align with sustainable agriculture principles and contribute to long-term food safety and security.

Essential oils (EOs) have gained increasing attention as promising alternatives to synthetic insecticides, primarily due to their biodegradability, reduced toxicity to non-target organisms, and rich composition of bioactive compounds [19,20]. These natural substances are complex mixtures of volatile secondary metabolites, such as terpenes, phenols, and aldehydes, synthesized by aromatic plants as part of their defense against herbivores and pathogens [21,22]. Their diverse biological activities—including insecticidal, repellent, antifeedant, and ovicidal effects—highlight their potential for inclusion in sustainable pest management programs [23,24].

Chile harbors a highly diverse native flora shaped by its unique geography and climatic range, from arid deserts to temperate rainforests. This botanical richness includes numerous native and endemic species known for producing bioactive secondary metabolites with potential applications in agriculture, medicine, and biotechnology [25,26,27,28,29]. Among these, EOs extracted from *Pilgerodendron uviferum* (Cupressaceae), *Laureliopsis philippiana* (Atherospermataceae), *Adesmia boronioides* (Fabaceae), and *Drimys winteri* (Winteraceae) have demonstrated insecticidal activity against agricultural pests and disease vectors [30]. *Haplopappus foliosus* (Asteraceae) EO also showed insecticidal activity against *Drosophila melanogaster* and interacted with acetylcholinesterase, suggesting a possible neurotoxic mechanism of action [29]. In this sense, *Drimys winteri* (J.R. Forst. & G. Forst.) commonly known as canelo, a tree species native to southern Chile and Argentina [31], exhibits interesting biological activities through its EOs. Recent studies have identified both bark- and leaf-derived EOs of *D. winteri* as rich sources of bioactive compounds with antifungal, antimicrobial, and insect-repellent properties [30,32,33,34]. These findings position *D. winteri* as a promising candidate for developing environmentally friendly pest control strategies.

This study aims to contribute to the development of safer and more sustainable pest management strategies by investigating the repellent potential of this native Chilean tree. Specifically, the repellent and oviposition deterrent activities of EOs extracted from *D. winteri* were assessed against *D. suzukii* using dual-choice bioassays. In addition, the chemical composition of these EOs was analyzed by gas chromatography–mass spectrometry (GC/MS).

## 2. Results

### 2.1. Chemical Profile of Drimys winteri Bark and Leaf Essential Oils

The GC/MS analysis (Table 1) enabled the identification of 21 compounds, accounting for 99.9% of the total relative abundance of the bark EO of *D. winteri*. The chemical profile was predominantly composed of monoterpenes, which represented 77.55% of the total composition. The major constituents were α-pinene (49.64%), followed by β-pinene (17.17%) and D-limonene (6.82%). Minor monoterpenes included 3-carene (1.62%) and p-cymene (1.86%). Sesquiterpenes accounted for 19.38% of the total composition. The most abundant compounds in this class were α-cubebene (6.75%) and β-cubebene (2.39%). Additional sesquiterpenes were detected in lower amounts, including γ-cadinene (1.30%), α- and γ-muurolene, santalene, caryophyllene, and oxygenated derivatives such as caryophyllene oxide and τ-cadinol, as well as drimenol (1.07%). Non-terpenoid compounds were represented by 2-piperizinopyridine, which accounted for 3.07% of the total relative area.

With respect to the leaf EO from *D. winteri*, a total of 31 compounds were identified, representing 98.63% of the total relative abundance. The chemical composition was also dominated by monoterpenes, which represented 69.69% of the total, with α-pinene (35.85%), β-pinene (17.61%), and D-limonene (6.77%) as the major constituents. Other monoterpenes, such as β-myrcene, α-phellandrene, γ-terpinene, and oxygenated derivatives including eucalyptol, borneol, terpinen-4-ol, and bornyl acetate, were detected in lower proportions. Sesquiterpenes accounted for 27.98% of the total composition, with elemol (7.07%), α-cubebene (2.21%), germacrene D (2.54%), and eudesmol isomers (α-, β-, and γ-eudesmol) among the most abundant compounds. Additional sesquiterpenes, including caryophyllene, humulene, cadinene derivatives, and spathulenol, were also identified in minor amounts. Diterpenes were present only in trace amounts (0.29%), represented by sandaracopimaradiene and α-kaurene. Non-terpenoid compounds and unidentified constituents accounted for 2.82% of the total relative area.

### 2.2. Choice Bioassay

**Figure 1:**
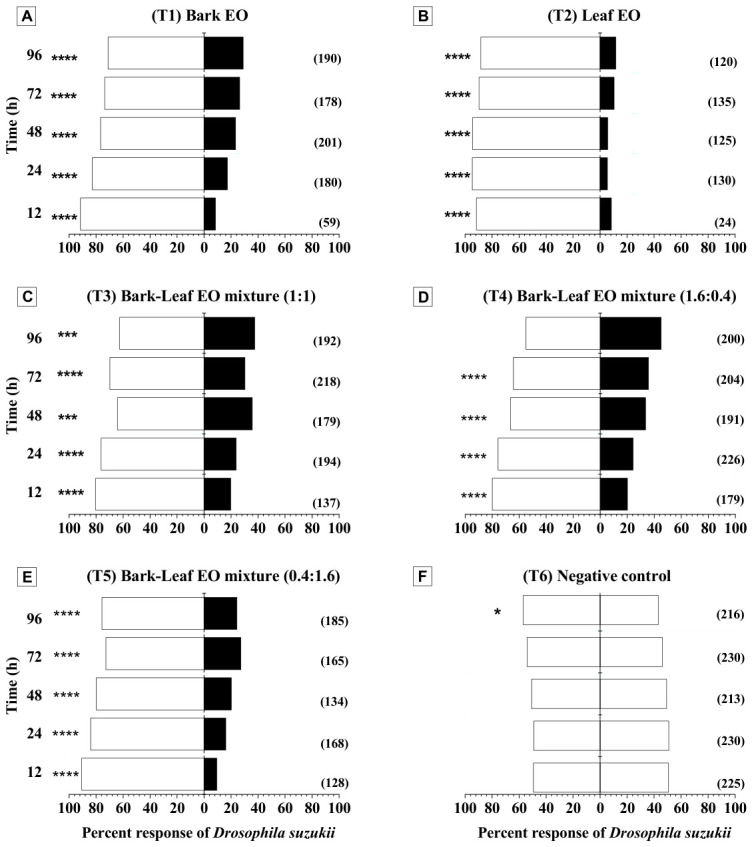
Percentage of *D. suzukii* choosing the control (white bars) or *D. winteri* EO treatments (black bars) in choice bioassays. The negative control contained acetone only. Bioassays were evaluated at 12, 24, 48, 72, and 96 h. Numbers in parentheses indicate the number of responding flies. Asterisks indicate significant differences based on *G*-tests: **** *p* < 0.0001, *** *p* < 0.001, * *p* < 0.05.

In bark EO (T1), 92% of flies chose the control at 12 h (*G* = 47.55, *df* = 1, *p* < 0.0001), 76% at 48 h (*G* = 60.01, *df* = 1, *p* < 0.0001), and 71% at 96 h (*G* = 34.76, *df* = 1, *p* < 0.0001). Heterogeneity among replicates was detected in all evaluation periods (*p* < 0.05) (Figure 1A).

In leaf EO (T2), the percentage of flies choosing the control ranged from 91% at 12 h (*G* = 19.50, *df* = 1, *p* = 0.00001) to 94% at 48 h (*G* = 119.33, *df* = 1, *p* < 0.0001), without heterogeneity among replicates (*p* > 0.05), and 88% at 96 h (*G* = 79.9, *df* = 1, *p* < 0.0001), with significant heterogeneity (*p* < 0.05) (Figure 1B).

The mixture of bark and leaf EOs (1:1) (T3) showed a similar trend to that observed with the oils separately, though to a lesser degree. The percentage of flies choosing the control ranged from 80% at 12 h (*G* = 53.93, *df* = 1, *p* < 0.0001) without significant differences across replicates (*p* > 0.05), to 63% at 96 h (*G* = 12.13, *df* = 1, *p* < 0.001) with significant heterogeneity (*p* < 0.05) (Figure 1C).

The mixture of bark and leaf EOs (1.6:0.4) (T4) showed a similar trend to that observed with the mixture 1:1. At 12 h, 80% of flies chose the control over the treatment (*G* = 68.45, *df* = 1, *p* < 0.0001), while at 72 h, it reached 64% (*G* = 16.72, *df* = 1, *p* < 0.001) with significant heterogeneity among replicates (*p* < 0.05). At 96 h, no significant preference was observed (*G* = 2.00, *df* = 1, *p* = 0.157) (Figure 1D).

In treatment 5 (T5), the proportions of bark and leaf EOs were reversed (0.4:1.6). At 12 h, 91% of flies chose the control (*G* = 97.80, *df* = 1, *p* < 0.0001), decreasing to 80% at 48 h (*G* = 51.10, *df* = 1, *p* < 0.0001) and 76% at 96 h (*G* = 51.19, *df* = 1, *p* < 0.0001). Heterogeneity among replicates was not detected at 12, 24, 48, and 96 h (*p* > 0.05), but was detected at 72 h (*p* < 0.05) (Figure 1E).

In the negative control (acetone vs. acetone) (T6), flies showed no side preference between 12 and 72 h, with pooled *G*-tests indicating no deviation from a 50:50 distribution (all *p* > 0.23). Although significant heterogeneity among replicates was detected (all *p*-values ≤ 0.015), no directional bias was observed, indicating the absence of positional effects in the assay. At 96 h, the proportion deviated from 50:50 (*p* = 0.041) (Figure 1F).

**Figure 2:**
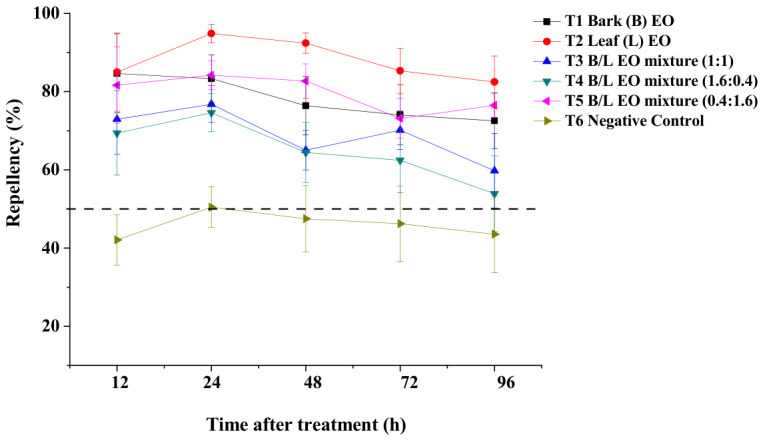
Repellency (%) of *D. suzukii* under different *D. winteri* EO treatments over time (12–96 h). Values represent mean ± SE. The horizontal dashed line at 50% indicates no preference for either stimulus.

All *D. winteri* EO treatments showed repellency percentages above 50% between 12 and 96 h (Figure 2, Table 2). The leaf EO (T2) showed the highest repellency, with values of 85.00 ± 10.00% at 12 h and 82.47 ± 6.63% at 96 h. However, no significant differences were detected between T2 and the other treatments (*p* > 0.05), except at 48 h, when it differed significantly from T3 and T4 (*p* < 0.05).

### 2.3. Oviposition Bioassay

The number of eggs laid on treated fruits with *D. winteri* leaf EOs was significantly lower than on control fruits at all concentrations tested. Significant differences were observed at 0.75 mg mL^−1^, with 2.76 ± 0.78 eggs in the treatment versus 6.68 ± 1.30 eggs in the control (Wilcoxon signed-rank test, *Z* = −2.37, *p* = 0.018); at 1.5 mg mL^−1^, with 0.64 ± 0.22 eggs in the treatment versus 11.32 ± 1.72 eggs in the control (*Z* = −4.05, *p* < 0.001); and at 3 mg mL^−1^, with 4.08 ± 0.98 eggs in the treatment versus 12.36 ± 1.84 eggs in the control (*Z* = −3.22, *p* = 0.001).

**Figure 3:**
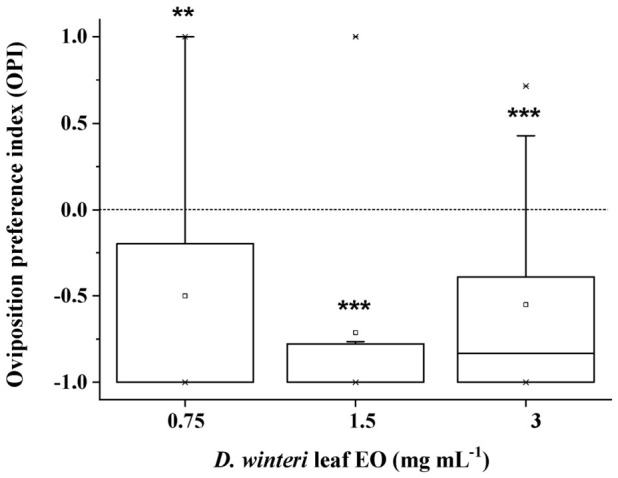
Oviposition preference index (OPI) of *D. suzukii* females exposed to raspberry fruits treated with *D. winteri* leaf EO (0.75, 1.5, and 3 mg mL^−1^). Negative OPI values indicate avoidance of treated fruits, whereas positive values indicate preference. The dashed line represents OPI = 0 (no preference). Asterisks indicate significant differences from zero based on Wilcoxon signed-rank tests (** *p* < 0.01, *** *p* < 0.001) and × indicates outliers. Each treatment included 25 independent replicates.

Oviposition preference index (OPI) was calculated (Figure 3). The OPI was significantly lower than zero at all tested concentrations (Wilcoxon signed-rank test: 0.75 mg mL^−1^, *Z* = −3.03, *p* = 0.002; 1.5 mg mL^−1^, *Z* = −3.91, *p* < 0.001; 3 mg mL^−1^, *Z* = −3.69, *p* < 0.001). The most negative mean OPI occurred at 1.5 mg mL^−1^ (−0.71 ± 0.11), followed by 3 mg mL^−1^ (−0.55 ± 0.11) and 0.75 mg mL^−1^ (−0.50 ± 0.14). No significant differences in OPI were detected among treatments (Kruskal–Wallis test, *p* = 0.118).

## 3. Discussion

In general, the bioactivity of EOs is linked to their chemical composition, which can vary even within the same species [35]. In this study, both bark and leaf EOs were dominated by monoterpenes, primarily α-pinene (49% in bark and 35% in leaf), followed by β-pinene (17% in both oils) and similar levels of D-limonene (~6.7%). Furthermore, the leaf EO exhibited greater chemical diversity than the bark EO, with more identified compounds (31 vs. 21) and a higher proportion of sesquiterpenes (28% vs. 19%). Among the sesquiterpenes identified in the leaf EO, elemol (7.07%) and the eudesmol isomers α-, β-, and γ-eudesmol (2.90%, 2.42%, and 2.95%, respectively) were noteworthy. Similar compounds were reported by Tampe et al. [32] in canelo leaf EOs from La Araucanía; however, in that study, these sesquiterpenes were the major compounds, whereas in the present study, α-pinene and β-pinene predominated.

Other studies conducted in different regions of Chile have also reported qualitative and quantitative differences in the EO chemical profiles of *D. winteri* [36,37,38,39,40,41]. For instance, Zapata et al. [37] described leaf EOs from south–central Chile as rich in γ-curcumene, limonene, myrcene, and trans-caryophyllene. In contrast, Becerra et al. [39] observed that oils from the Biobío Region were dominated by benzocycloheptene. Similarly, Muñoz et al. [41] found that monoterpenes predominated in leaf EOs from Chiloé Island populations, whereas sesquiterpenes and phenylpropanoids were more abundant in samples collected from continental areas near Santiago. Together, these findings highlight the chemical variability of *D. winteri* EOs and suggest that environmental and genetic factors may contribute to the occurrence of distinct chemotypes [34].

To date, no studies have evaluated the bioactivity of *D. winteri* EOs against *D. suzukii*. However, canelo EOs have been reported to exhibit repellent, insecticidal, and deterrent activity against insect pests [30,32,37,38]. In the present study, *D. suzukii* showed a clear preference for the control over raspberry jelly pots treated with *D. winteri* EOs, indicating an avoidance response to the treatments. The leaf EO (T2) produced the highest choice response toward the control, followed by the bark EO (T1) and the bark–leaf EO mixture (0.4:1.6) (T5), with more than 90% of flies choosing the control at 24 h and approximately 72% at 96 h. Lower choice responses were observed for the other bark–leaf EO mixtures (1:1) (T3) and (1.6:0.4) (T4), with approximately 80% at 24 h and 55% at 96 h. Similar avoidance responses have been reported by Renkema et al. [42], who observed variable levels of preference for untreated controls depending on the EO tested against *D. suzukii*.

Based on these results, the repellency percentage for each treatment was determined over time. T2 maintained the highest repellency throughout the evaluation period (85% at 12 h to 82% at 96 h), suggesting a more persistent repellent effect than the other treatments. Although T1 and T5 also showed high initial repellency, their effectiveness declined more rapidly. In contrast, T3 and T4 exhibited the lowest repellency. Similar observations were reported by Renkema et al. [42,43], who indicated that peppermint oil (*Mentha* × *piperita*) maintained 100% repellency against *D. suzukii* females up to 144 h after application, while geranium oil (*Pelargonium asperum*) showed a progressive decline in repellency, reaching approximately 35% at 96 h. In addition, Souza et al. [16] reported strong avoidance responses (~90%) in choice olfactometry assays, reflecting an immediate behavioral response of *D. suzukii* to the evaluated oils.

Olfactory cues are involved in oviposition site selection in *D. suzukii* [44], and exposure to aversive compounds can reduce this activity [45,46,47]. *D. winteri* leaf EO showed a deterrent effect under laboratory conditions, with negative Oviposition Index (OPI) values recorded at all evaluated concentrations. Egg deposition on treated fruits was reduced, and no significant differences were observed among concentrations, indicating comparable deterrent effects among them. Similar reductions in oviposition have been reported for other EOs against *D. suzukii*, including *Baccharis* spp. [48] and the EOs of *Citrus reticulata* and *Melaleuca alternifolia* [49], although the response varied among oils and concentrations. In our study, the 1.5 mg mL^−1^ treatment resulted in the lowest oviposition, with an average of 0.64 ± 0.22 eggs in treated fruits and 11.32 ± 1.72 eggs in control fruits. However, these differences were not statistically significant, possibly due to variability in the data. De Souza et al. [48] also found that the EOs of *B. calvescens*, *B. mesoneura*, and *B. oblongifolia* reduced oviposition on artificial fruits (≈7.6 eggs fruit^−1^) compared with the water (17.2 eggs fruit^−1^) and acetone (17.6 eggs fruit^−1^) controls.

The greater repellent and oviposition-deterrent activity observed may be associated with the chemical profile of the leaf EO. The main compounds identified, α-pinene and β-pinene, have been previously associated with insecticidal and oviposition-deterrent effects against *D. suzukii* in studies using *Baccharis* spp. EOs [48]. In addition, D-limonene has been identified as one of the most effective compounds associated with repellent responses in this species during choice and olfactory assays [50]. At the same time, EO bioactivity is not determined exclusively by major compounds, but also by interactions among constituents, including synergistic and antagonistic effects [19,35,51]. The canelo leaf EO had a greater proportion of sesquiterpenes than the bark EO (27.9% vs. 19.4%), including germacrene D, elemol, and the eudesmol isomers (α-, β-, and γ-eudesmol), none of which were detected in the bark EO. Although present in lower amounts than the dominant monoterpenes, these sesquiterpenes may contribute to EO bioactivity. Information regarding the effects of these sesquiterpenes against *D. suzukii* remains limited; however, Chu et al. [52] reported β-eudesmol as toxic to *Drosophila melanogaster*, suggesting that these compounds may also contribute to the behavioral responses observed in the present study.

## 4. Materials and Methods

### 4.1. Insects

The *D. suzukii* colony used in the bioassays was maintained in BugDorm insect-rearing cages (MegaView Science Co., Ltd., Taichung, Taiwan) at 22 ± 1 °C, 60% relative humidity, and a 12:12 h (L:D) photoperiod. Flies were provided with water through moist cotton and fed a modified artificial diet as described by Renkema et al. [43]. The diet consisted of agar, cornmeal, sugar, nutritional yeast, propionic acid, methyl paraben dissolved in 95% ethanol, and raspberry pulp.

### 4.2. Drimys winteri Essential Oils and Chemical Analysis

EOs from *D. winteri* bark and leaf were obtained by Campestre (Temuco, Chile), through steam distillation for 1.5 h from plant material collected in the La Araucanía Region, Chile. Each EO was analyzed with GC/MS (Thermo Focus GC, Thermo Fisher Scientific, Waltham, MA, USA). Each EO was diluted in hexane at 1 µg µL^−1^, and 1 µL of each one was injected, separately, into a capillary column BPX5 (30 m length, 0.25 µm film thickness, and 0.25 mm inner diameter). The operating conditions are described in Tampe et al. [32]. The Xcalibur software version 4.2.47 (Thermo Fisher Scientific Inc., Waltham, MA, USA) was used to acquire and process data. The compounds were identified by comparing their mass spectra with a library database (NIST ver. 2.0, NIST, Gaithersburg, MD, USA) and by comparing their Kovats indices with those reported in the literature [53,54].

### 4.3. Experimental Arena and Setup

The bioassays were conducted in clear acrylic boxes (37 cm long × 23 cm high × 23 cm wide) equipped with tight-fitting lids and white mesh on all four sides to ensure adequate ventilation. Each experimental arena contained two plastic cups positioned 25 cm apart. All assays were conducted in a climate-controlled room at 22 ± 1 °C, 60% relative humidity, and a 12:12 h (L:D) photoperiod.

#### 4.3.1. Choice Bioassay

The bioassays were conducted to evaluate the behavioral response of *D. suzukii* to different *D. winteri* EO treatments. In each experimental arena, two plastic cups (150 mL) containing 15 g of raspberry jelly were used as an attractant for adult flies. The jelly consisted of agar (2 g), fructose (5 g), distilled water (200 mL), raspberry pulp (175 mL), and methyl paraben stock solution (2 mL). A cotton wick was placed in each cup to apply the treatments. The cotton wick received 2 mL of pure acetone (control), while the other received 2 mL of an EO solution diluted in acetone to a final concentration of 15 mg mL^−1^. Five treatments were evaluated: (T1) bark EO, (T2) leaf EO, (T3) a 1:1 (*v*/*v*) mixture of bark and leaf EOs, (T4) a bark: leaf EO mixture (1.6:0.4, *v*/*v*), and (T5) a bark:leaf EO mixture (0.4:1.6, *v*/*v*). Before each assay, the cups were placed under an extraction hood for 1 h to allow solvent evaporation. Subsequently, 25 unsexed adult flies (3–5 days old) were released into the center of the arena and allowed to move freely toward either cup. The number of flies present on each cotton wick (treatment or control) was recorded at 12, 24, 48, 72, and 96 h after release. To verify the absence of positional bias within the arena, choice assays were conducted using identical treatments on both sides. In these assays, 2 mL of pure acetone was applied to the cotton wick of each cup. Ten independent replicates were performed for each treatment and the negative control.

#### 4.3.2. Oviposition Bioassay

The bioassays were conducted to evaluate the deterrent oviposition effect of *D. winteri* leaf EO on female *D. suzukii*. Based on the previous results, the leaf EO was selected because it showed the highest repellency over time. In each experimental arena, two natural raspberry fruits (cv. Heritage) were used as oviposition substrates. Raspberries were harvested before the trial to ensure firmness and uniform size. Three EO concentrations were tested (0.75, 1.5, and 3 mg mL^−1^). A volume of 500 μL of each EO solution diluted in acetone was applied to one fruit, while 500 μL of pure acetone was applied to the control fruit. Both treated and control fruits were kept for 1 h under a laminar flow hood to allow solvent evaporation. Each fruit was then placed inside a plastic cup (30 mL). In each arena, one treated and one control fruit were presented simultaneously, and 10 female and 5 male adult *D. suzukii* (5 days old) were released. After 24 h, the flies were removed, and the number of eggs laid on each fruit was counted under a stereoscopic magnifying glass (7–45× zoom). Each concentration was replicated twenty-five times.

**Figure 4:**
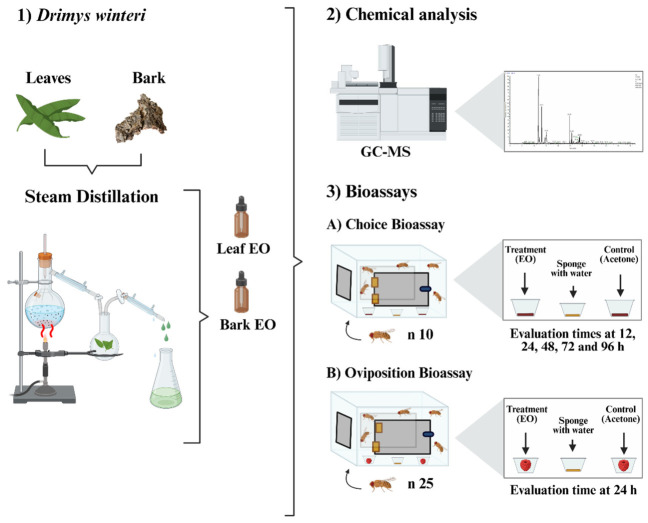
Experimental workflow of the study, including EO extraction, chemical characterization, and choice bioassays conducted with *D. suzukii*. The figure was created with BioRender.com.

The experimental workflow used in this study is shown in Figure 4.

### 4.4. Data Analysis

The statistical software SPSS version 26 (IBM Corp., Armonk, NY, USA) was used for data analysis. Before analysis, data were subjected to the Shapiro–Wilk test to assess normality. Since the data were not normally distributed, non-parametric tests were used. In the choice bioassay, the distribution of flies between the control and treated wicks was used to assess choice behavior. Choice data were analyzed in two complementary stages. First, goodness-of-fit tests (*G*-tests) were performed for each treatment to determine whether flies showed a significant preference for the treated stimulus over the control. When responses were consistent across replicates (heterogeneity test, *p* > 0.05), pooled G statistics were calculated. Repellency percentages were calculated for each replicate as shown in Equation (1), where C represents the number of individuals recorded in the control and T the number of individuals recorded in the treatment.

Repellency (%) = [C/(C + T)] × 100(1)

Repellency values were compared among treatments using the Kruskal–Wallis test, followed by Bonferroni-adjusted pairwise comparisons when significant differences were detected.

For the oviposition bioassays, the oviposition preference index (OPI) was calculated as shown in Equation (2), where T represents the number of eggs laid on the treated substrate and C the number of eggs laid on the control substrate.

OPI = (T − C)/(T + C)(2)

Differences in the number of eggs laid between treated and control substrates were evaluated using Wilcoxon signed-rank tests for paired samples, performed separately for each concentration. Differences in OPI among treatments were analyzed using the Kruskal–Wallis test. Additionally, Wilcoxon signed-rank tests were conducted for each treatment to determine whether OPI values differed from a neutral response (OPI = 0).

All figures were generated using OriginPro 2024b (64-bit), version 10.1.5.132 (OriginLab Corporation, Northampton, MA, USA).

## 5. Conclusions

*D. winteri* EOs, particularly the leaf EO, showed repellent and oviposition-deterrent activity against *D. suzukii* under laboratory conditions. These effects may be linked to the leaf EO’s chemical profile, which is dominated by monoterpenes and contains a higher proportion of sesquiterpenes than the bark EO. The reduced attraction and oviposition on treated substrates suggest that the leaf EO may support the development of alternative pest management strategies. However, further studies are needed to identify the compounds responsible for the observed behavioral responses and to evaluate their efficacy and persistence under field conditions.

## Figures and Tables

**Table 1:** Chemical composition of *Drimys winteri* bark and leaf essential oils.

Compound	RT	RI	Bark (%)	Leaves (%)	Identification
α-Pinene	11.85	937	49.64	35.85	RI, MS
2-Piperizinopyridine	12.20	-	3.07	1.45	MS
β-Pinene	13.17	979	17.17	17.61	RI, MS
β-Myrcene	13.96	991	-	1.84	RI, MS
α-Phellandrene	14.25	1005	-	2.05	RI, MS
3-Carene	14.46	1011	1.62	-	RI, MS
β-Cymene	14.88	1023	1.86	-	RI, MS
Eucalyptol	14.95	1032	-	0.99	RI, MS
D-Limonene	15.08	-	6.82	6.77	MS
γ-Terpinene	16.14	1060	-	1.18	RI, MS
(+)-2-Bornanone	18.32	1143	0.27	-	RI, MS
(−)-Borneol	19.29	1166	-	0.20	RI, MS
Terpinen-4-ol	19.95	1177	-	0.98	RI, MS
(−)-Bornyl acetate	22.71	1284	0.11	0.18	RI, MS
Elemene isomer	24.37	1344	-	0.45	RI, MS
α-Cubebene	24.76	1351	6.75	2.21	RI, MS
α-Copaene	25.46	-	2.49	0.53	MS
β-Cubebene	25.76	1389	2.39	-	RI, MS
β-Elemene	25.81	1391	-	1.48	RI, MS
β-Maaliene	26.32	1405	0.34	-	RI, MS
Caryophyllene	26.51	1419	0.34	1.60	RI, MS
Santalene	26.58	1420	0.49	-	RI, MS
β-Copaene	26.80	1432	-	0.24	RI, MS
Cadina-3,5-diene	27.26	1458	-	0.22	RI, MS
Humulene	27.35	1454	-	0.39	RI, MS
γ-Muurolene	27.90	1477	0.39	-	RI, MS
Germacrene D	28.00	1481	-	2.54	RI, MS
Bicyclogermacrene	28.37	1495	-	1.06	RI, MS
α-Muurolene	28.47	1499	0.72	-	RI, MS
γ-Cadinene	28.77	1513	1.30	-	RI, MS
Cadina-1(10),4-diene	28.96	-	2.26	1.95	MS
Elemol	29.49	1549	-	7.07	RI, MS
Spathulenol	30.21	1576	-	0.23	RI, MS
Caryophyllene oxide	30.29	1581	0.57	-	RI, MS
γ-Eudesmol	31.42	1631	-	2.95	RI, MS
τ-Cadinol	31.75	1640	0.20	-	RI, MS
β-Eudesmol	31.80	1649	-	2.42	RI, MS
α-Eudesmol	31.94	1653	-	2.90	RI, MS
Drimenol	34.32	1761	1.07	0.89	RI, MS
Cembrene	37.84	1939	-	0.12	RI, MS
Sandaracopimaradiene	38.18	1960	-	0.02	RI, MS
α-Kaurene	39.89	2041	-	0.16	RI, MS
Monoterpenes	-	-	77.5	69.69	-
Sesquiterpenes	-	-	19.38	27.98	-
Diterpenes	-	-	0	0.29	-
Others	-	-	2.98	1.36	-

RT—retention time (min); RI—Kovats retention index; %—considering detected compounds; MS—mass spectra. Compounds written in bold correspond to the most abundant compounds detected in bark and leaf EOs from *D. winteri*.

**Table 2:** Repellency (%) of *D. suzukii* in response to different treatments (T1–T6) over time (12–96 h).

Time (h)					
	12	24	48	72	96
T1	84.67 ± 10.09 ^a^	83.29 ± 6.12 ^a^	76.38 ± 7.46 ^ab^	74.06 ± 7.70 ^ab^	72.56 ± 7.15 ^a^
T2	85.00 ± 10.00 ^a^	94.85 ± 2.35 ^a^	92.40 ± 2.62 ^a^	85.30 ± 5.81 ^a^	82.47 ± 6.63 ^a^
T3	72.94 ± 9.00 ^ab^	76.81 ± 4.73 ^ab^	65.00 ± 5.07 ^b^	70.15 ± 4.90 ^ab^	59.77 ± 9.52 ^a^
T4	69.42 ± 10.77 ^ab^	74.62 ± 4.84 ^ab^	64.45 ± 7.67 ^b^	62.44 ± 8.28 ^ab^	53.90 ± 9.71 ^a^
T5	81.66 ± 9.74 ^a^	84.19 ± 3.69 ^a^	82.73 ± 4.37 ^ab^	73.18 ± 5.04 ^ab^	76.49 ± 3.07 ^a^
T6	42.08 ± 6.40 ^b^	50.46 ± 5.16 ^b^	47.50 ± 8.49 ^b^	46.22 ± 9.58 ^b^	43.53 ± 9.78 ^a^

Values are shown as mean ± SE (n = 10). Different letters within the same column indicate significant differences among treatments based on the Kruskal–Wallis test followed by Bonferroni multiple comparisons. T6 represents the negative control.

## Data Availability

The original contributions presented in this study are included in the article. Further inquiries can be directed to the corresponding authors.
